# Supplementary material for: Are carbon emissions trading and green financial instruments synergistic? -Comprehensive quantitative research based on content analysis
Source: PLoS One. 2024 Mar 7;19(3):e0298601. doi: 10.1371/journal.pone.0298601 (PMC10919601; doi:10.1371/journal.pone.0298601)
Supplement: S1 Data — (ZIP) [file pone.0298601.s001.zip › Data/地级市Z_F工作报告.zip!/╣π╬≈/╖└│╟╕█╩╨/└┤╫╘╣1⁄2╓┌║┼í╛┬φ┐╦╩2╛▌═°í┐_2010.docx]

各位代表：

现在，我代表市人民政府向大会作政府工作报告，请予审议，并请各位政协委员和其他列席会议的同志提出意见。

2009年工作回顾

2009年是极不平凡的一年，是我市积极应对国际金融危机，刻苦攻坚、逆势而上、跨越发展的一年。在自治区党委、政府和市委的正确领导下，全市上下深入学习实践科学发展观，紧紧抓住国家扩大内需和广西北部湾经济区发展规划深入实施等重大机遇，以“率先构筑沿海发展新高地，倾力打造中国东盟合作第一城”为主题，把“促增长、增投资、扩内需、调结构、抓城建、上产业”作为首要任务，深入实施“项目建设攻坚年”和“服务企业年”，大力推进“刻苦攻坚跨越发展三年计划”、“九大工程”和“双百工程”，全市经济实现危机之年“速度不慢、增幅不低、排位不降”，各项工作成效显著，社会和谐稳定，边境稳固安宁，较好地完成了市四届人大五次会议确定的目标任务。

全市经济逆势高扬，主要指标高居榜首。全市生产总值完成243.8亿元（初步统计，下同），增长22.6%，连续三年增幅保持20%以上，不仅继续排全区各市首位，而且进入全国地级市前列，成为我国经济增长最快、最具活力的城市之一。规模以上工业增加值等7项指标增幅和港口货物吞吐量等6项指标总量高居全区各市之首。“十一五”规划24项经济指标有15项提前达到，其中人均GDP达到4180美元。

项目建设高潮迭起，投资劲增业绩非凡。举全市之力扎实推进钢铁、核电两大“天字号工程”和产业发展、城市建设“双百工程”，实现“月月有开工，季季有竣工”。全社会固定资产投资完成254.1亿元，增长74%，增速排全区第一，是“十五”期间的两倍。投资对经济增长的贡献率超过80%。“双百工程”项目在建246个，竣工42个，完成投资197.4亿元。争取到中央扩大内需项目和资金为历年之最。钢铁、核电两大项目完成投资30.9亿元，钢铁项目主厂区陆域平整、吹沙填海及防浪墙工程基本完成，江山半岛休疗养中心开工建设；核电项目进厂道路建成通车，1、2号机组核岛负挖和准备工程全面完成，推进速度创造了中国核电建设历史新纪录。两大项目都已具备开工条件。中冶实久、中一重工从开工到投产分别仅用了12个月和14个月。重交沥青、大海粮油三期技改等重大产业项目和崇左至钦州高速公路防城港段等重大基础设施项目开工建设。全国百强企业、大型有色金属冶炼企业金川集团落户我市的前期工作稳步推进，防城至东兴高速公路等项目前期工作加快。上思华润水泥、海港500KV变电站等项目基本完工。

按照市委部署，完善征地搬迁政策和工作机制，组织两千多名干部进村入户，共征地3万多亩，报批获准新增建设用地、用海1.4万亩，盘活、收储土地1.3万亩，为项目建设提供了有力保障。“文明征地，和谐搬迁”的防城港模式，得到中央部委和自治区党委、政府的充分肯定，作为典型经验在全区推广。

服务企业成效显著，工业经济全面飚升。及时出台扶持政策，重点帮助企业解决资金、市场、运力等困难，使多数企业较快地走出困境，实现工业经济良性运行。规模以上工业总产值实现342.2亿元，增长23.4%；增加值97.6亿元，增长39.8%。工业技改投资完成26.8亿元，增长144%，是“十五”期间的6.8倍。工业经济效益综合指数排全区第一，同比提高74.8个百分点。全市规模以上工业企业达到143家，产值超30亿元的工业企业达到3家，强优工业企业数为广西北部湾经济区各市之最。企沙工业区、大西南临港工业园、粮油食品产业园和东兴边境经济合作区纳入广西北部湾经济区重点产业园区布局规划，企沙工业区、防城工业园区、上思工业集中区被批准为自治区A类产业园区，结束了我市没有A类产业园区的历史。园区基础设施完成投资5.1亿元。入园投产企业70家，产值占全市规模以上工业的83.5%。

城市建设亮点纷呈，港城面貌日新月异。出台《促进防城港市城市建设暂行办法》等一系列激励政策，以“四沿线、四片区、两景观、两改造”为重点，大力推进城市建设百项工程，掀起了前所未有的城市建设热潮。全市“满城是工地，到处有项目”，在建项目达125个，竣工22个，完成投资75.6亿元。2008年以来，城市建成区面积从32平方公里增加到42平方公里。中心区和桃花湾南部片区城市格局基本形成，城南新区开发启动，城市主框架全面拉开，“一年有所变化”目标已经实现。城市路网建设大规模推进，兴港大道、友谊路路面改造竣工，风貌立面改造全面启动；北部湾大道改造一期工程、东兴大道维修改造完成；行政中心区路网、桃花湾南部片区路网全面开工，部分路段已建成通车；北部湾大道改造二期、珍珠大道等10多条城市道路加快建设。城市功能不断增强，北部湾广场主体工程、仙人山公园一期建成开放；市污水处理厂投入使用；青少年活动中心等四大场馆、城投公司综合楼等一批大型建筑开工建设；德城大酒店等星级宾馆和世界风情步行街等商业项目抓紧实施。桃花湖公园等一批城市景观项目加快推进，中央商务区等城市标志性建筑的论证工作基本完成。“城在海中、海在城中”的全海景生态滨海城市初见端倪。完成房地产投资51.3亿元，增长74.1%，金海湾、阳光海岸等一批房地产项目全面建设，商品房销售面积增长46.8%。城市总体规划修编和土地利用总体规划获自治区批复实施，开展城市专项规划27个。强化城市风貌管理，重视城建项目单体设计审查。城乡清洁工程深入实施，城市净化、美化、亮化成效显著。东兴市、上思县新城区建设和旧城改造建设取得新进展。

港口经济逆势发展，现代物流增势强劲。全面贯彻“以港兴市”战略，首次召开全市加快港口建设动员大会，出台加快建设国际枢纽大港的意见，对港口发展进行新的战略布局，港口发展进入了新的历史阶段。完成港口码头设施建设投资17.5亿元，增长67.7%。18—22号泊位水工工程和东、西湾航道工程已完工，403—407号泊位和20万吨级进港航道工程等项目加快推进。在受金融危机影响的不利形势下，全市港口货物吞吐量完成6380万吨，占广西沿海港口近7成，防城港港务集团完成4500万吨，增长21.6%，增速远高于全国港口平均水平，提前一年达到“十一五”规划目标。全市物流总体规划及加油站布点规划编制完成。东湾物流园建设加快推进，中外运仓储物流基地等项目抓紧实施，东湾大道等路网工程全面动工。全市新增物流企业25家。广西首个电子口岸海运物流服务平台开通运行，通关效率大幅提升。全社会货运量8600万吨，增长23.3%；货物运输周转量207.3亿吨公里，增长45%。

“三农”工作扎实有效，县域经济实力增强。农业加快向特色化、规模化和专业化方向发展，粮食实现增产；种植“红姑娘”红薯、金花茶等“红金系列”特色农作物18万亩和糖料蔗55万亩；建立一批无公害养殖示范点，广西第一个大型深水抗风浪网箱养殖项目在我市投产成功；制定了扶持水产品加工业等政策，促进农业龙头企业发展壮大。集体林权改革加快，完成外业勘界面积325.7万亩，造林18.6万亩。完成病险水库除险加固、农村饮水安全等一批农村基础设施项目。第三批贫困村整村推进、兴边富民行动和水库库区移民基础设施建设大会战扎实推进，全年脱贫7000多人。县域经济发展迅速，生产总值158.3亿元，增长18.4%。防城区、东兴市被评为广西科学发展十佳县，港口区、上思县分别被评为广西招商引资工作先进县和广西科学发展进步县，实现“县县先进县县红”。

财政金融运行良好，融资实现历史突破。在国家实施结构性减税和调整边贸税收政策，全市重点税源减收4亿元的情况下，实行综合治税，狠抓增收，迅速扭转财政收入1月份负增长41.8%的不利局面，实现逐月提升，全年财政收入完成27.4亿元，增长24.9%，提前一个月完成年度任务。以保增长、惠民生为重点的财政支出进度快速有效，完成40.2亿元，增长53.5%。支出结构不断优化，一般公共服务支出得到有效控制。对政府项目进行投资评审，核减资金3.8亿元。出台鼓励银行业支持地方发展的奖励办法，新增金融机构贷款71.2亿元，增长2.8倍，提前3个月完成自治区下达任务。建行、工行在我市恢复二级分行，广西北部湾银行防城港支行成立，率先创办了广西北部湾经济区第一家村镇银行——东兴国民村镇银行。充分发挥城投公司等四大市级融资平台的作用，整合资源，解决融资担保问题，政府性融资贷款到位资金36.9亿元，创历史新高，是2008年的8倍。与建行、中行、国开行共签订了授信金额1100亿元的政银合作协议。争取到自治区搭桥贷款8亿元解决项目资本金问题。采取BT等方式融资建设一批市政设施。通过转贷方式支持县（市、区）项目建设。融资运作成功为大开发大建设提供了前所未有的强大资金保障。

旅游商贸日趋兴旺，第三产业快速发展。按照市委打造“国际滨海旅游胜地”和“中国海洋文化名城”的总体要求，明确了十大旅游区发展思路，出台扶持旅游业发展的优惠政策，全面启动旅游文化基础设施和景区景点建设大会战，开工建设41个项目，完成投资10亿元。全市旅游发展总体规划批复实施，14个景区规划编制完成，理顺了江山半岛旅游度假区管理体制。“十个一”旅游文化品牌项目扎实推进，大型海上实景演出《印象·北部湾》已经启动，“一首防城港之歌”《腾飞防城港》已经开唱。十万大山国家森林公园已获国家旅游局批准为4A级景区，京岛风景名胜区创4A通过自治区初检。魔石谷、簕山古渔村等一批新景区相继建成。出台扶持宾馆建设等优惠政策，万海、彼岸国际等一批星级宾馆先后开业。旅游大篷车等宣传推介活动卓有成效。全年接待国内游客418.1万人次，增长87.8%；旅游收入17.1亿元，增长91.3%。住宿、餐饮、交通等传统服务业快速发展；信息服务、规划设计等现代服务业日趋兴旺。开工建设汇通·卓富商贸城、东兴东盟商贸广场等10多个商贸市场。“万村千乡市场工程”建设加快，全市商业网点达2.8万个。城乡消费稳中趋旺，全社会消费品零售总额45.3亿元，增长18.8%。

区域合作力度加大，沿边开放国际好评。以打造“中国东盟合作第一城”为目标，对东兴边境经济合作区重新审视、谋划新策，全力推进沿边沿海开放工作。成功举办2009中越边民友好大联欢活动，获得中越两国领导人的高度好评。成功举办首届国际龙舟节、京族哈节等重大节庆活动，在国内外反响强烈。落实国家促进边贸发展政策，迅速建成东兴新边民互市贸易区一期工程、里火边民互市贸易区并封关运行，边民互市的规模和档次迈上新台阶，全市边境贸易成交额112.3亿元，增长21.9%。外贸进出口总额21.7亿美元，进口总额排全区第一。参加第六届中国—东盟博览会等重大会展，与越南共同举办越中（芒街—东兴）国际商贸·旅游博览会，签订合作项目投资总额140多亿元。引进区外境内资金90亿元，增长105%。在境外设立办事机构或分公司15个，企业“走出去”为广西各市之首。与韩国永同郡等友好城市的交流进一步密切。先后组团出访东盟、日韩、欧洲及港澳台等地，引起各地高度关注，提高了知名度。

民生工作高层肯定，社会事业全面进步。在减收因素多、可用财力十分有限的情况下，2009年市财政对民生的投入达到8亿元，增长1倍，占全市财政收入的近三分之一，是民生投入最大、解决民生问题最多的一年。在全国率先实施孤儿养育新标准，出台了系统全面的救助制度，被评为“2009年度中国社会政策十大创新”之一，得到民政部充分肯定，我市作为唯一地市代表在全国民政工作会议上作典型发言。在全国率先建立弱势群体档案，逐一落实帮扶政策。在全区率先将重大项目被征地农民纳入城镇低保和城镇职工养老保障范围。提高了城乡低保、公益性岗位工资补贴、村干部补贴标准和社会养老、医疗保险待遇水平。被征地群众安置工作扎实推进，北港住宅区首期22栋公寓楼、大平坡住宅区27栋安置房已交付使用，大龙住宅区正在加快建设。新增城镇就业23688人，安置钢铁、核电项目被征地农民就业2492人。市政府承诺为民办的9件实事已经完成。人民生活水平显著提高，城镇居民人均可支配收入1.6万元，增长11.9%；农民人均纯收入5000元，增长11.6%。

各项社会事业加快发展。市高级中学扩建工程、职业教育中心开工建设。引进了80多名高级专业人才，是建市以来引才层次最高、数量最多的一年。群众文化活动丰富多彩,出版了防城港市文化遗产名录丛书等书籍，东兴京族博物馆正式开馆，建成首批农家书屋98个。全民健身广泛开展，市桃花湾体育馆开工建设，建成187个村标准篮球场。创“三甲”医院工作扎实推进，市第一人民医院门诊医技楼落成使用，市中医院住院楼开工建设；城镇职工基本医疗保险参保率85%，新型农村合作医疗参合率95.9%，超额完成自治区下达任务；实施全民健康行动，为6万多参保人员提供免费健康体检；甲型H1N1流感防控工作积极有效，全市无死亡病例发生。与自治区科技厅建立了科技工作会商机制，四个县（市、区）全部通过全国县（市）科技进步考核，东兴市、防城区荣获“全国科技进步考核先进县（市）”称号。建成计生服务站所26个，东兴市被评为“全国计划生育优质服务先进单位”，人口自然增长率控制在计划指标范围内。

信访、调处和应急工作扎实开展，社会治安大整治专项行动成效显著。安全生产事故起数、伤亡人数全面下降，被评为“全区安全生产优秀单位”。渔洲坪磷化企业等环境整治力度加大，节能减排得到加强，主要污染物排放量控制在自治区要求范围内。推进政府投资项目竣工审计，核减资金4300万元。完成我市第二次全国经济普查。物价水平得到有效控制。海关、检验检疫、海事、气象等部门服务水平提升。“双拥”创建力度加大。市场监管、国资监管、边海防、侨务、司法、宗教、民族、人防、档案、修志、妇女儿童、残疾人、防震减灾等各项工作取得新成效。

政府建设全面加强，服务水平显著提升。“行政高效能项目建设无腐败”行动深入实施。依法行政全面推进。电子政务系统开通运行。制定实施《关于加强科学民主决策提高行政执行力的意见》，对政府工作人员进行执行力大培训，促进政府执行力明显提升。开展建市以来规模最大的一次文件清理工作，共清理政府规范性文件642件，废止和修改了一批不适宜的政策文件。出台《关于加快项目前期工作的若干意见》等规范性文件，完善了行政审批、招投标等40多项政策制度。自觉接受市人大和市政协监督，共办理并答复市人大代表提出的意见、建议76件，办复率100%；办理并答复市政协委员提案127件，办复率100%。完善项目联审制和手续代办制，开设大项目行政服务“直通车”，开展市长接待日等活动，加强现场对接、现场协调，审批效率明显提高。强化行政问责，开展百人评议机关活动；开通行政效能投诉“绿色通道”，为企业提供“保姆式”服务，机关效能显著增强。企业对我市投资环境连续两年达到高度满意区间。

各位代表！

2009年是我市面临困难最多、挑战最大、任务最艰巨的一年。全市上下共克时艰，只争朝夕、废寝忘食地干，“白加黑”、“5加2”地干，“人一之我十之，人十之我百之”地干，经济社会多项指标继续领先全区，创造了辉煌业绩，为实现“十一五”规划目标奠定了坚实基础，极大地增强了全市各族人民的信心和决心。取得如此骄人成绩，我们深刻体会到：一是必须紧紧依靠市委的坚强领导，坚决贯彻市委决策，积极应对前进道路上的各种困难，做到审时度势、超前谋划、敢于攻坚、善于冲刺；二是必须强化政府执行力，特事特办、急事急办，早布置、快协调，勤检查、严问责，确保工作不折不扣地落到实处；三是必须坚持改革开放，大胆创新体制机制，及时出台刺激经济增长的政策措施，为发展创造良好环境；四是必须坚持重大项目带动战略，突出临港工业、城市建设、港口、旅游等重点，着力破解项目建设、投融资、征地搬迁和边贸发展等瓶颈难题，带动全局发展；五是必须坚持以人为本，认真解决群众最直接最现实的利益问题，竭力改善民生；六是必须充分调动各方面的积极性，放开思想想问题、放开手脚干事业，形成推进工作的强大合力。

成绩来之不易，经验弥足珍贵。这是自治区党委、政府和市委正确领导的结果，是市人大、市政协有效监督、大力支持和共同努力的结果，是中直、区直单位和驻军驻警支持帮助的结果，是全市各族人民万众一心、艰苦创业的结果。在此，我谨代表市人民政府，向全市广大干部群众，向解放军指战员、武警官兵和公安民警，向各民主党派、工商联、无党派人士、人民团体，向所有关心、支持、参与我市现代化建设的各界人士和海内外朋友，表示衷心的感谢和崇高的敬意！

在总结成绩的同时，我们也清醒地认识到，我市经济社会发展还存在不少问题，主要表现为：主导产业带动力不强；受外需不足影响，出口增长乏力；基础设施仍然滞后，城市软实力和综合竞争力有待提升；投资环境仍需改善；群众关心的一些热点难点问题还没有得到根本解决，不稳定因素依然存在。对这些问题，市政府将在今后工作中高度重视，努力加以解决。

2010年工作目标和主要任务

今年是实施“十一五”规划和“刻苦攻坚跨越发展三年计划”的最后一年，也是中国—东盟自由贸易区正式建成的第一年。当前，国际金融危机对经济发展的不利影响依然存在，我市经济运行形势依然严峻，面临诸多不确定因素。一是钢铁项目尚未获得国家正式核准，一大批上下游产业和配套项目未能落户,投资保持超高速增长十分困难；二是我市尚未进入广西北部湾经济区保税物流体系，港口优势弱化，发展遇到前所未有的挑战；三是工业经济缺乏新的主导产业，工业高速增长难以为继。在直面困难的同时，更要看到有利条件。目前全球经济加快复苏，我国经济企稳向好势头明显，国家进一步扶持广西经济社会发展的力度前所未有，自治区实施北部湾经济区优先发展战略的力度空前加大，全市经济实力不断增强，工业化、城镇化进程加快，大建设、大发展的格局已经形成，全市上下干事创业的热情高涨。总体上看，今年的经济形势是好的，有利条件多于不利条件，机遇大于挑战。只要我们提振信心，鼓足干劲，把基础打牢，把后劲攒足，坚持不懈地干下去，就一定能够把握新机遇、应对新挑战、创造新辉煌！

根据市委部署，今年市人民政府必须以科学发展观为指导，按照市委“速度要快、效益要好、排位不降、民生要上”的要求，紧紧抓住国务院出台关于进一步促进广西经济社会发展的若干意见和中国—东盟自贸区建成等重大机遇，以“项目建设突破年”、“民生改善年”、“环境优化年”为主题，以“快增长、扩投资、抓城建、上产业、拓开放、惠民生”为主攻方向，坚持“稳中求增抓一产，增中求快促二产，快中更快提三产”，着力转变经济发展方式，加快发展临港工业和第三产业；着力增强投资拉动，提升产业发展和城市化水平；着力优化环境，推进开放合作；着力改善民生，保持社会和谐稳定，促进经济社会又好又快发展，为早日建成新兴港口工业城市、重要门户城市和国际枢纽大港、中国海洋文化名城而努力奋斗。

全市经济社会发展的主要预期目标是：地区生产总值增长17%，财政收入增长17%，全社会固定资产投资增长30%，全部工业增加值增长16.2%，社会消费品零售总额增长22%，外贸进出口额增长15.4%，实际利用外资增长25%，港口货物吞吐量增长20%，城镇新增就业人数2万人，城镇登记失业率控制在4.5%以内，人口自然增长率控制在11‰以内，万元地区生产总值能耗、化学需氧量排放总量、二氧化硫排放总量控制在自治区要求范围内。

以上目标，与我市实施“十一五”规划和全面建设小康社会要求相衔接，体现了科学发展、和谐发展、跨越发展的要求，有利于增强信心、稳定预期。在不确定因素增加的形势下，实现经济增长17%是相当不容易的，需要付出艰苦努力才能实现。

在实际工作中，我们将朝更高目标努力，通过强化政府执行力，全面落实自治区优先发展北部湾经济区的工作部署，做到“谋划更早，布置更快，推进更实，效果更好”，集中力量、打破常规，施展大手笔、采取大动作，努力实现“大产业、大港口、大交通、大物流、大城建、大旅游、大招商、大文化”的重点突破。具体抓好以下十项工作：

一、抓好三个“天字号工程”，加快打造临港大产业

继续举全市之力，抓好防城港钢铁基地、红沙核电、金川铜镍大型冶炼项目三个“天字号工程”。以大产业为根本，通过重大项目带动产业链发展，培育壮大临港产业集群。

全力推进防城港钢铁基地项目。加强沟通协调，完善前期工作，争取国家早日核准开工。抓好主厂区及配套产业区、安置区的基础设施建设。加快钢铁配套码头、航道、厂区道路、休疗养中心等工程建设。开工建设指挥部办公楼、职工宿舍等配套项目。

全力加快红沙核电项目建设。争取上半年获得国家核准，全面开工建设。加快施工准备区和核电生活基地建设。年内力争完成投资30亿元以上。

全力推进金川铜镍大型冶炼项目。做好选址规划和项目可研，完成用地用海申报，3月份开展征地拆迁工作，力争上半年开工建设，三年内一期项目建成投产。

全力促进临港工业有效聚集。加大帮扶企业力度，确保工业经济良性运行。支持大海粮油、惠禹蛋白、盛隆冶金、中华电力、中一重工、中冶实久等重点企业发展。加快重交沥青、盛农磷酸深加工等在建项目建设。开工建设枫叶菜籽油等项目。抓好华润水泥厂一期投产及二期建设。着力推动食品、磷化、机械、冶金、建材和修造船等行业发展。加快嘉里粮油和惠禹蛋白小包装技改、盛隆冶金结构调整、东兴鉴声液晶彩电生产线等一批技改项目建设，完成技改投资46亿元以上。

二、实施“项目建设突破年”，掀起新一轮投资热潮

以“项目建设突破年”为主题，以产业发展和城市建设“双百工程”为重点，注重投资效益，全年完成全社会固定资产投资330亿元以上。

强力推进园区建设。把六大产业园区作为投资的突破口和主要平台，突出抓好自治区重点支持的企沙工业区和大西南临港工业园，打造以钢铁、有色金属为龙头的现代化国际工业区和以磷化产业、资源加工型企业为主的大西南出口加工基地。积极争取自治区各类专项资金，实施园区基础设施项目37个，年度计划投资16.7亿元。企沙工业区要基本建成钢铁上下游配套主干路网，迅速建成金川有色金属加工园进厂道路。大西南临港工业园要全面开工园区路网工程，主干道路基本贯通，开工建设工业污水处理和沙港安置区配套设施，全年完成工业产值超100亿元。粮油食品产业园上半年要完善园区路网工程，整治园区环境，全年完成工业产值超175亿元。东兴边境经济合作区要完成园区置换和新区规划工作，动工建设国际商贸物流园等项目。防城工业园区和上思工业集中区要抓好园区供水扩建、路网和污水、垃圾处理等项目。

强力推进大交通建设。构建以大交通为基础的综合交通体系。加快崇左至钦州高速公路、钦防铁路扩能改造、南防快速铁路建设。开工建设防东高速公路、滨海公路防城港段、钦防高速公路扩建工程。贵台至防城二级公路防城港段、沙企一级路企沙连接线年内完工。加快市道路运输信息管理指挥中心建设，结合高速铁路客运站规划建设城市交通枢纽中心。开展防城至东兴铁路前期工作。

强力推进产业大招商。以大招商聚产业，把招商引资作为经济工作的生命线工程，依托六大产业园区，健全市县（市、区）两级招商引资联动机制、财税共享机制和项目滚动发展制度，组织专业招商团队，实行组合式招商、点对点招商。加强与东盟国家和地区的交流，加大与区内外大型国企的战略合作。发展“飞地经济”，加快承接产业转移，吸引一批实力强的央企、外企参与或独资建设若干专业产业园或“园中园”。利用好防城港务集团等企业品牌，创新以企招商模式，拓宽产业招商渠道。全年引进区外境内资金108亿元以上，利用外资1.5亿美元以上，实现招商引资新突破。

强力破解项目建设瓶颈。加大投入，完善机制，扎实推进一批事关长远、影响巨大的产业和基础设施项目前期工作。加快编制“十二五”规划和专项规划。按照“重组架构、增加力量、明确分工、加强协调”的要求，抓好征地搬迁安置工作，妥善处理历史遗留问题；加大闲置土地清理和用地用海报批力度，保障建设用地需要。坚持项目联审和规划土地预审，继续推行市长接待日等行之有效的做法，加快项目审批。强化项目管理，做到业主、设计、施工、监理到位，坚持管理机构、施工设备、施工措施、监督检查到位，确保项目进度快、质量好。

强力提升投融资水平。充分发挥城投公司等四个市级融资平台的作用，落实1100亿元的政银合作协议，进一步拓宽融资渠道。围绕“双百工程”建立融资项目库，提高项目成熟度。加强金融服务，破解企业融资难题。发挥民营经济在房地产、商贸物流和旅游业等领域中的主力军作用。

三、以大城建为核心，构筑百万人口城市主框架

突出“滨海、门户、生态”三大特色，按照“三岛三湾一核心六组团”的总体布局和“四沿线、四片区、两景观、两改造”的建设重点，继续推进城市建设百项工程，打好“八个一批”攻坚战，努力完成投资130亿元以上，确保建一片成一片，倾力打造全海景生态滨海城市。

建设一批城市主干道。上半年完成北部湾大道二期改造工程、西湾大道和珍珠大道建设。按城市道路标准完成东兴大道改造。江山大道西段基本贯通，东湾大道全线开工。

建设一批城市标志性建筑。启动以高层建筑群为主的中央商务区建设，打造城市核心区。基本建成科技图书馆、博物馆、文化艺术中心、青少年活动中心、城投公司综合楼主体工程。加快东盟外滩建设。

建设一批“四沿线、四片区”项目。全面清理“四沿线”用地，限期开发沿线地块。基本建成商业中心区和桃花湾北部片区路网。全面动工建设城南新区路网。加快各片区房地产、商业设施、生活服务设施等城市配套项目建设，完善组团功能。

建设一批西湾景观中心项目。全面开工建设西湾整治、西湾景观大道、针鱼岭大桥等项目，加快针鱼岛、洲墩岛的整理和规划工作，推进红林海湾等临湾建筑建设。

建设一批便民利民项目。建成桃花湖公园一期工程，推进仙人山公园、蜈蚣岭公园、白鹭公园、体育公园建设。加强小区物业管理，开展文明小区评比。规划建设停车场、集贸市场、公交车站等一批市政配套设施，提升城市服务功能。

建设一批房地产项目。金海湾商住区一期年内基本完工，海悦华府、汇通·卓富商贸城、宝来大厦、中环花园等项目实现竣工，开工建设西湾华庭、越秀实业、华港丽城等项目，加快阳光海岸、800里上城等项目建设，推动房地产业有序发展。

实施一批旧城改造项目。加快港口区烂尾楼处置和城中村改造，启动渔洲坪工业园整体搬迁工作；抓好桃花湖周边村改和冲孔安置区建设。加快防城区汽车站整体搬迁，推进商业中心与休闲广场建设，完成民族大道、珍珠大道风貌改造。

推进一批县城和重点镇建设。上思县城南和城东新区主干路网工程年内建成，扩大城区规模，凸显森林旅游胜地风貌。东兴市实施“东扩北移”，推进国门广场和口岸改造项目，9月底完成国门广场主通道建设。企沙镇以北港、大龙安置区为重点，加快建成企沙工业区配套生活基地。

四、以大港口为龙头，做优做强大物流

坚持“以港兴市”战略，全面落实市委建设国际枢纽大港的战略决策，以大港口为龙头，做强大物流；以大物流为支撑，壮大港口经济，实现跨越发展、率先崛起。

全力打造大港口。今年港口货物吞吐量要完成7600万吨，早日建成亿吨大港。年内建成防城港18—22号泊位，加快建设20万吨级进港航道、东湾403—407号泊位，开工建设运通、信润码头等工程，启动企沙工业区配套码头、潭油作业区5万吨级航道、防城港40万吨减载平台及配套航道前期工作。抓好茅岭港等中小港口的建设和管理。完成防城港总体规划修编。强化海岸线利用和管理，合理规划港区及配套服务设施。抓好还岛于港，为港口发展留足空间。全面扩大港口对外开放，采取多种合作方式，加强与周边城市、东盟各国港口的合作，吸引航运业巨头参与港口建设。增开直航世界主要港口的航线，发展远洋运输。实施奖励政策，重点扶持集装箱运输和航运业发展壮大。支持港口企业拓展市场。建成港务集团东大门，实施港区封闭管理，推进人货分流。加快防城港口岸联检大楼、港航商务大厦建设。切实帮助防城港务集团解决历史遗留问题。

奋力发展大物流。充分发挥大港口优势，规划建设矿石、煤炭配送中心。做好煤炭物流基地项目前期工作。积极争取设立保税物流中心、保税物流园区等海关特殊监管区。完成东湾物流园区主干路网建设，加快园区土地清理、环境整治工作及在建仓储物流项目建设。开工建设冲仑物流园区道路，推进大型停车场、租赁市场和仓储配送项目。制定优惠政策，扶持物流企业做大做强，打响“防城港物流”品牌。完善货物运输信息化管理。完善口岸通关、办公和生活等设施，提高通关效率和服务质量。

五、切实加强“三农”工作，发展壮大县域经济

认真贯彻落实中央一号文件精神，按照“稳粮保供给，增收惠民生，改革促统筹，强基增后劲”的要求，努力提高统筹城乡发展能力，进一步夯实农业发展基础。

优化农业结构。积极应对中国—东盟自贸区建成对我市农业带来的机遇和挑战，大力发展生态农业、品牌农业、休闲农业等现代农业。抓好粮食生产，确保粮食安全。做大做强“红姑娘”红薯、红龙果、红莲雾等“红系列”和金花茶、金香糯等“金系列”特色农业。发展生态健康养殖，稳步推进深水抗风浪网箱养殖，扩大罗非鱼、对虾和生猪、家禽养殖规模。加大对甘蔗、金花茶、油茶、水产品、香料、林板等农林产品加工业的扶持。

深化农村综合改革。抓好集体林权制度改革，完成160万亩的林改任务。加快基层农技体制和十万山华侨林场体制改革。抓好整合村级综合服务设施试点村建设。积极推广完善“农事村办”等新型服务模式，提高农村社会化服务水平。

强化农业基础设施建设。加快以水利、道路等为重点的农村基础设施建设，完成病险水库除险加固22座、中越界河整治10公里、防渗加固三面光渠道50公里，新建标准堤防10公里，恢复和改善灌溉面积30万亩。实施土地整治整村推进工程。加快黄竹村、牛路村等新农村示范点建设。完成第三批贫困村整村推进工作，建成一批村屯道路、饮水工程、沼气池等项目。推进边境重大动物疫病防控阻截带建设。

增强县域经济竞争力。落实市委《关于加强县域经济重点工作的若干意见》，大力发展县域特色经济。港口区重点依托钢铁、核电等重大项目发展临港临海产业；防城区重点打造重大项目的配套产业基地和后勤保障基地；上思县重点发展绿色产业、资源加工业和特色旅游业；东兴市要完成东兴组团规划，统筹好产业发展和城市建设，重点发展商贸、旅游、物流及出口加工业。

六、大力发展现代服务业，打造国际旅游商贸热点城市

以大旅游为增长点，更加重视发展第三产业，努力搞旺人气、繁荣市场、促进消费、提升生活品质。

加快发展大旅游。近期建成广西旅游热点城市，全面实施“十个一”旅游文化品牌项目和建设国际滨海旅游胜地行动方案，以十大旅游景区为主战场，掀起旅游文化基础设施和景区景点建设大会战热潮，开工建设重点项目70个，新增投资80亿元以上。完成京岛风景名胜区创4A景区工作，加快十万大山森林公园4A景区升级改造。江山半岛旅游度假区游客接待中心、停车场等景区配套设施“五一”节前建成开放。抓好北仑河口跨国生态旅游区建设。规划建设旅游客运码头、邮轮专用港和游艇基地。完善簕山古渔村等一批生态旅游专业村，大力发展农家乐、自驾游。今年要在国内主要媒体滚动播出旅游宣传片，继续开展旅游大篷车活动，制作沿路大型旅游宣传牌，采取多种方式进一步扩大旅游宣传促销。加强旅游行业监管和旅游从业人员培训，加快景区道路建设，力争德城五星级大酒店等一批接待设施年底竣工，全面提升旅游服务水平。

加快发展商贸业。完善商业网点规划，引进和培育一批大超市、快餐连锁店、品牌商贸企业。世界风情步行街一期“五一”节前开业，抓紧建设东兴旅游购物街。围绕增强片区功能，在商业中心片区开工建设购物商场、建材市场、花卉市场等商贸设施；在桃花湾片区开工建设金海商贸城、越秀百货超市，建成桂海·东盟新城大型商场、农贸市场。积极推进企沙中国—东盟钢材交易配送中心、东兴进出口机电综合市场等一批专业市场建设。抓好“家电下乡”等工作，繁荣城乡商贸业。

加快发展其他服务业。大力发展信息服务、金融保险、规划设计、中介咨询等现代服务业。积极贯彻落实国家促进消费政策，扩大住房、汽车、体育健身、休闲娱乐、住宿餐饮等热点消费。

七、进一步扩大对外开放，打造中国东盟合作第一城

抓住中国—东盟自贸区建成的机遇，全面推进与东盟各国的开放合作，加快将我市打造成中国东盟合作第一城。

积极发展对外经济。利用自贸区零关税和国家鼓励政策，开拓东盟市场,重点抓好粮油、机电、化工、煤炭、矿石等大宗商品进出口。加快边贸转型，争取设立东兴、茅岭进口资源加工区，规划建设水产品、矿产品等加工贸易基地和橡胶、红木家具等专业市场。充分利用港口和边境口岸优势，构建对接东盟投资与服务的通道和前沿基地，建设机械设备和物料配送中心。

积极扩大区域合作。以中越建交60周年为契机，与越南等东盟国家在商贸、物流、农业、旅游、文化、会展等领域开展全方位合作。加强与越方对接，积极推进中国东兴—越南芒街跨境经济合作区规划和申报工作。力争动工建设北仑河二桥。加快东兴、里火边民互市贸易区二期工程建设；启动杨屋、滩散互市贸易区规划建设；完善企沙边贸码头基础设施，改善通关环境。重点拓展与西南地区、长三角、珠三角、港澳台地区及友好城市的合作交往。积极组织参加上海世博会、中国—东盟博览会等重大会展活动。支持企业“走出去”，加强国际经济技术合作。

八、完善财政运行机制，促进经济与社会协调发展

深化财政体制改革，充分发挥财政职能，着力培育支柱财源，调整和优化支出结构，集中财力办大事，提高财政管理绩效。

狠抓增收节支。抓好重大项目、重点企业、重点行业的税源培育，积极扶持发展第三产业，努力开拓新财源。加快财政收入结构优化调整，提高税收收入比重。严格控制一般性支出，继续加大财政对社会事业、民生保障、生态环保等方面的投入，构建公共服务型财政。

强化财政扶持作用。建立“放水养鱼”机制，设立产业发展基金，加大财政对港口、城建、旅游、水产品加工等领域的扶持。抓好中小企业融资担保工作。

推进财政管理体制改革。按照财权与事权相匹配的原则，加快理顺市区两级财政管理体制。完善综合预算编制，健全国库管理制度。提高财政投资评审水平，加强政府融资贷款管理，提高资金使用的规范性和有效性。完善偿债机制，积极防范财政风险。配合做好自治区直管县财政管理体制改革试点工作。

九、实施“民生改善年”，构建幸福和谐防城港

以民生福祉为根本，加大投入，继续实施事关民生的全民健康行动、实现充分就业行动、提高全民素质行动、增强发展责任行动，提升城市形象和文化品位，努力把我市建成全区民生改善最好、人民收入增长最快、幸福指数最高的宜居城市之一。

实施更加积极的就业政策。完善就业服务体系,抓好职业技能培训和职业介绍工作，促进全民创业。重点对3300名被征地农民进行就业培训。抓好下岗失业人员、被征地农民、边境居民、城乡“零就业家庭”等群体的就业工作。

健全城乡居民社会保障体系。抓好新型农村社会养老保险试点，实现3万人参保。基本解决破产企业、困难企业职工和退休人员参加医疗保险问题。进一步提高城镇职工基本医疗保险参保率和新型农村合作医疗参合率。抓好被征地农民安置和社会保障工作。规范城乡低保管理，提高保障标准。认真落实孤儿救助、老年人优待等政策,健全弱势困难群体档案，提高社会救助和社会福利水平。

优先发展教育科技事业。继续实施农村初中改造、寄宿制学校建设和校舍安全工程。完成市高级中学扩建一期和市职业教育中心一期工程。力争开工建设高等职业技术学院。新建一所市级特殊教育学校。推进与科技厅的深度合作，组织实施一批科技项目；深化产学研合作，为重点产业、重点企业发展提供科技支撑。

倾力发展大文化。更加重视发展文化产业，以大文化树形象，提升城市软实力。《印象·北部湾》大型实景演出力争10月首演，完成一台原创舞台戏、一部电影、一套综合反映防城港市情况的丛书等工作。努力办好京族哈节、国际龙舟节、金花茶节、十万大山森林旅游节、中越（东兴—芒街）国际商贸·旅游博览会等重大文化旅游节庆活动，扩大影响力。积极举办或承办各种重大会议、重要论坛，繁荣会展经济。抓好公共文化服务和文化市场体系建设，丰富广场文化，鼓励建设社区影视院、社区文化活动中心、图书专业市场和农村文化活动室。深入开展文化下乡活动，抓好第二批农家书屋建设。加强新闻出版工作和地域文化、京、瑶等民族特色文化的保护开发。认真贯彻《全民健身条例》，开展群众性体育运动。建成市桃花湾体育馆，推进社区和农村体育设施建设。完成796个20户以上已通电自然村“村村通”广播电视工程。

深化医药卫生体制改革。加强公共卫生体系建设，提高疾病预防控制能力。突出抓好市第一人民医院、市中医院创“三甲”工作，完成市中医院住院楼主体工程，开工建设市精神病医院和市第一人民医院新住院大楼。完善农村三级卫生服务网络，发展社区卫生事业。

做好人口计生工作。组织开展我市第六次全国人口普查。抓好计生服务站建设。积极开展诚信计生和“两无一提高”创建活动。加大财政投入和奖扶力度，为计划生育家庭办实事、办好事。

创建国家环境保护模范城。启动国家环境保护模范城创建工作。完成生态功能区划编制。大力发展生态产业和循环经济，抓好上思蔗糖综合利用循环经济试点和生物质发电项目。加强江河流域综合治理，加大环境污染整治力度。深入开展“百万农户种千万棵树”活动，掀起新一轮造林绿化热潮，积极创建国家森林城市。强化节能减排责任，确保完成自治区下达的任务。东兴、上思污水处理厂6月底竣工投入使用。开工建设市垃圾处理厂技改项目。全市城镇污水集中处理率和垃圾无害化处理率均达到60%以上。

建设和谐防城港。深入开展社会治安整治行动，严厉打击黑恶势力和“两抢两盗”、走私、贩毒等犯罪活动。强化安全生产，遏制重特大事故发生。加强应急管理平台和队伍建设，有效应对各种突发事件。全面完成“五五”普法。加大法律援助。建立健全矛盾纠纷调处机制，促进社会安定团结。

抓好精神文明建设等工作。推进边海文明示范带、文明社区等群众性文明创建工作。加强网络文化建设和管理。改善少数民族生产生活条件，促进各民族团结繁荣。加强国防教育、国防动员、人民防空和民兵预备役建设，积极为部队做好生活保障和服务。深入开展“双拥”工作，认真落实军人优待抚恤政策，抓好军转干部、复退军人和随军家属安置。关心和支持老龄、妇女儿童、残疾人工作。依法管理宗教事务。加强市场监管，推进“质量兴市”建设。边海防、对台、档案、修志、气象、机关事务、防震减灾等各项工作都要取得新的成绩。

努力为民办好一批实事。在全面落实自治区为民办实事项目的基础上，结合我市实际，集中财力办好一批事关民生的实事：

1.为农村独生子女户和双女结扎户进行一次免费体检，并建立健康档案。

2.完成贫困残疾人危房改造400户。

3.完成边境3-20公里兴边富民行动基础设施建设大会战，改造1282户危房、修建150公里村屯道路、架设126公里农村电网。

4.解决5万农民的饮水难问题和2500户农村卫生改厕问题。

5.建设桃源路小学、金海湾小学、北港小学。

6.实施廉租房保障3716户；新建廉租住房652套，总建筑面积3.26万平方米。

7.实施城乡风貌改造工程，集中改造一批旧城区、城中村和“竹筒房”，全面推进一批交通要道、城镇、村庄的风貌改造。

8.新建6个城市垃圾中转站和6个公厕，建设40个公交站点，增开2条公交线路。

9.对港口、防城两城区90条小街小巷进行硬化、美化、亮化，增设排水排污管道。

十、实施“环境优化年”，建设人民满意政府

以实施“环境优化年”为契机，加强政府自身建设，全面提升政府执行力和服务水平，不断优化政务环境和投资环境。

强化政府执行力。政府及各部门必须真抓实干，措施有力，责任到位，确保市委决策迅速准确地落实到位，确保重大工作强势推进、限期完成。进一步落实《关于加强科学民主决策提高行政执行力的意见》，狠抓执行力的提升。认真落实首问负责制等“三项制度”，有事马上办，有问题马上协调，将“马上就办”作为政府机关工作人员的行动准则。强化奖惩激励制度，搞好绩效考评。认真落实问责制，深入开展行政效能监察“责任追究大行动”，加强督查督办，形成一级抓一级、层层抓落实的局面。

强化政府服务。开展“审批大提速和机制大健全行动”，减少审批环节，优化审批程序，创新审批机制，真正实现“直通车”、“保姆式”服务。加强政务服务中心建设，完善企业挂牌服务制度，协调解决企业面临的问题和困难。加强电子政务建设。进一步扩大政务公开。

深化政府机构改革。认真贯彻中央、自治区精神，实行大部门体制，理顺部门职责关系，上半年完成市、县两级政府机构改革任务。加快理顺城市管理体制，明确市本级与城区事权，规划权高度集中，建设权统分结合，管理权尽量下放。进一步完善工业园区管理体制，为产业发展和城市建设创造良好的体制机制环境。

加强民主法制建设。自觉接受人大、政协监督，广泛听取各方面意见。认真办好人大代表意见、建议和政协委员提案。充分发挥工会、共青团、妇联等群团组织的作用。巩固和扩大基层民主。加大依法治市力度，全面推进依法行政。加强廉政建设，深入开展“行政高效能项目建设无腐败”活动，打造廉洁高效、求真务实的人民满意政府。

各位代表!

回顾2009年，我们克难攻坚，成就斐然。展望2010年，机遇与挑战并存，任务艰巨、责任重大。让我们紧密团结在以胡锦涛同志为总书记的党中央周围，依靠市委的坚强领导，动员广大干部群众团结奋战，以钢铁般的意志、核能般的动力，树立百倍信心，鼓起万分勇气，迎难而上，乘势而为，努力开创我市科学发展、和谐发展、跨越发展的新局面！
